# Pulsed field ablation as a bailout for ventricular tachycardia ablation: a case report

**DOI:** 10.1093/ehjcr/ytaf437

**Published:** 2025-09-12

**Authors:** Laurens Verhaeghe, Geoffroy Ditac, Romain Tixier, Pierre Jaïs, Frédéric Sacher

**Affiliations:** Department of Cardiac Pacing and Electrophysiology, Hopital Cardiologique du Haut-Leveque, Bordeaux University Hospital, Avenue Magellan, 33600 Pessac, Bordeaux, France; IHU Liryc, Electrophysiology and Heart Modeling Institute, University Bordeaux, 33600 Pessac, Bordeaux, France; Department of Cardiology, Jessa Hospital, 3500 Hasselt, Belgium; Department of Cardiac Pacing and Electrophysiology, Hopital Cardiologique du Haut-Leveque, Bordeaux University Hospital, Avenue Magellan, 33600 Pessac, Bordeaux, France; IHU Liryc, Electrophysiology and Heart Modeling Institute, University Bordeaux, 33600 Pessac, Bordeaux, France; Department of Cardiac Pacing and Electrophysiology, Hopital Cardiologique du Haut-Leveque, Bordeaux University Hospital, Avenue Magellan, 33600 Pessac, Bordeaux, France; IHU Liryc, Electrophysiology and Heart Modeling Institute, University Bordeaux, 33600 Pessac, Bordeaux, France; Department of Cardiac Pacing and Electrophysiology, Hopital Cardiologique du Haut-Leveque, Bordeaux University Hospital, Avenue Magellan, 33600 Pessac, Bordeaux, France; IHU Liryc, Electrophysiology and Heart Modeling Institute, University Bordeaux, 33600 Pessac, Bordeaux, France; Department of Cardiac Pacing and Electrophysiology, Hopital Cardiologique du Haut-Leveque, Bordeaux University Hospital, Avenue Magellan, 33600 Pessac, Bordeaux, France; IHU Liryc, Electrophysiology and Heart Modeling Institute, University Bordeaux, 33600 Pessac, Bordeaux, France

**Keywords:** Ventricular tachycardia, Ablation, Pulsed field, Intramural substrate, Septal substrate, Case report

## Abstract

**Background:**

Catheter ablation of ventricular tachycardia (VT) using radiofrequency energy has proven to be an effective therapy. The outcome in patients presenting with an intramural septal substrate has however been disappointing. New tools and energy such as pulsed field ablation (PFA) might improve our efficacy to treat arrhythmia in this setting.

**Case summary:**

A 64-year-old patient with valvular and ischaemic cardiomyopathy had recurrence of arrhythmic storm despite previous endocardial ablation and stereotactic body radiation therapy targeting the interventricular septum. A new attempt using PFA via a novel large footprint 9 mm lattice-tip catheter allowed to achieve non-inducibility. Early recurrence of VT however occurred, necessitating a second procedure targeting zones that were ablated in the previous procedure. Careful analyses of the first procedure highlighted three possible technical reasons for this recurrence, which were taken into account during the redo ablation. At 7-month follow-up, the patient remained free from VT.

**Discussion:**

Endocardial ablation applying pulsed field energy through a lattice-tip catheter was capable of successfully treating an intramural septal substrate in a patient with multiple prior failed ablation procedures. Extra attention should be paid to specific properties of PFA for VT to avoid recurrences.

Learning points

- Pulsed field ablation using a novel large footprint lattice-tip catheter was capable of ablating a complex intramural substrate.
- An optimal ablation protocol with this catheter for ventricular tachycardia ablation still has to be determined.
- This case highlights the importance of ablation repetitions per site and appropriate catheter to tissue contact to reach the intramural substrate.

## Introduction

Catheter ablation (CA) for the treatment of ventricular tachycardia (VT) in structural heart disease has been proven an effective therapy.^1–3^ Results in patients presenting with a septal substrate are however worse.^4^ Catheter ablation using pulsed field energy (PFA) is a new ablation modality, where electrical fields are applied to the cells, leading to a disruption in cell membrane integrity and eventually cell death.^5^

We report on a patient who underwent successful PFA targeting a septal substrate for recurrent VT after failed ablation with radiofrequency energy (RFA) and stereotactic body radiation therapy (SBRT).

## Summary figure

**Table:** 

Time point	Event
*Another centre*	
1997	Ross procedure because of severe aortic insufficiency due to a bicuspid valve. Complicated reimplantation of the right coronary artery, necessitating a venous bypass graft to the right coronary artery. Periprocedural infarction of the right ventricle.
2012	Mechanical Bentall procedure because of recurrence of severe aortic insufficiency.
2022	Implantation of a cardiac resynchronisation therapy defibrillator(CRT-D).
February 2024	First endocardial VT ablation because of VT storm with conventional radiofrequency energy. One VT successfully ablated at the inferoseptal base of the left ventricle. A second VT had an intramural isthmus located in the apicoseptal region, which was targeted from the right and the left ventricle, but without success.
	Stereotactic radioablation of the apicoseptal part 1 week after the endocardial ablation.
	Recurrence of ventricular storm 6 days after stereotactic radioablation, treated medicinally.
	Refused for a left ventricular assist device and for heart transplantation because of comorbidities.
March 2024	CRT-D shock.
June 2024	CRT-D shock.
*Our centre*	
August 2024	Recurrence of VT storm with multiple CRT-D shocks. Left ventricular ejection fraction 20%.
	Second endocardial ablation. Big inferoseptal scar, four VTs induced, all with a probable septal isthmus. Ablation of the septum and inferior wall with pulsed field energy based on activation mapping and local abnormal ventricular activation. Non-inducible.
September 2024	Recurrence of VT storm with a slow VT at 107 beats per minute.
	Third endocardial ablation. Isthmus of the VT at the basal anteroseptal part of the RV, where previous ablations had already been performed. Second VT with an isthmus at the inferior apical part of the RV, where previous ablations had as well been performed. Ablation of both regions with pulsed field energy. Patient non-inducible.
April 2025	No recurrence of VT.

## Case presentation

A 64-year-old male patient with a cardiac resynchronization therapy defibrillator (CRT-D) because of valvular and ischaemic cardiomyopathy underwent an endocardial VT ablation with RFA because of arrhythmic storm in February 2024 in another centre. A VT originating in the apicoseptal region could not be eliminated, for which he received SBRT 1 week later. An early recurrence with VT storm was treated with anti-arrhythmic drug therapy. Two more VT episodes necessitated a CRT-D shock during a 4-month follow-up. In August 2024, he was admitted at our hospital after multiple appropriate defibrillator shocks despite a treatment with bisoprolol and amiodarone.

Upon arrival, he was hemodynamically stable with a paced ventricular rhythm. Echocardiography showed biventricular dilatation with a left ventricular ejection fraction of 20%. A cardiac computed tomography (CCT) scan with late contrast enhancement showed an extensive septal substrate.

**Figure 1:**
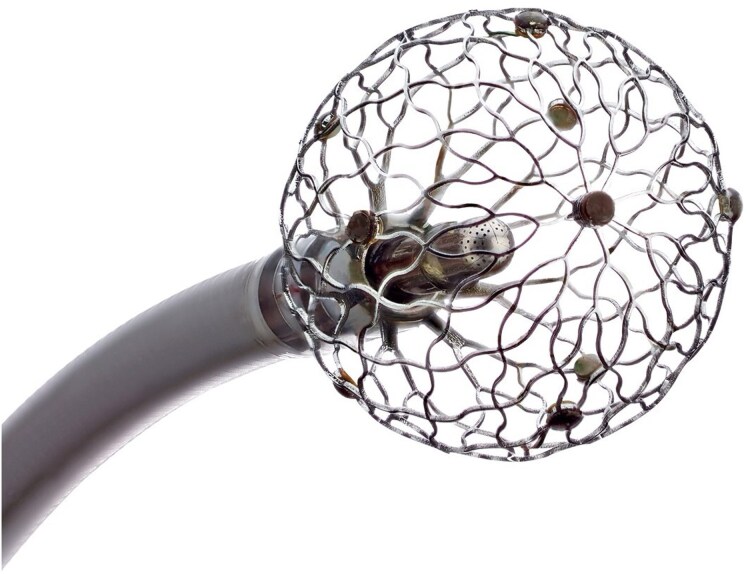
Image of the Sphere-9 catheter. It consists of a lattice-tip with nine surface electrodes: one tip electrode, four distal electrodes, and four proximal electrodes. Mapping as well as ablation can be performed with this catheter. It has a large footprint with a diameter of 9 mm, where usual standard solid tip catheters have a tip of 3.5 mm. Radiofrequency as well as pulsed field energy can be delivered through this catheter. The image is used with the permission of Medtronic. © 2024 Medtronic.

**Figure 2:**
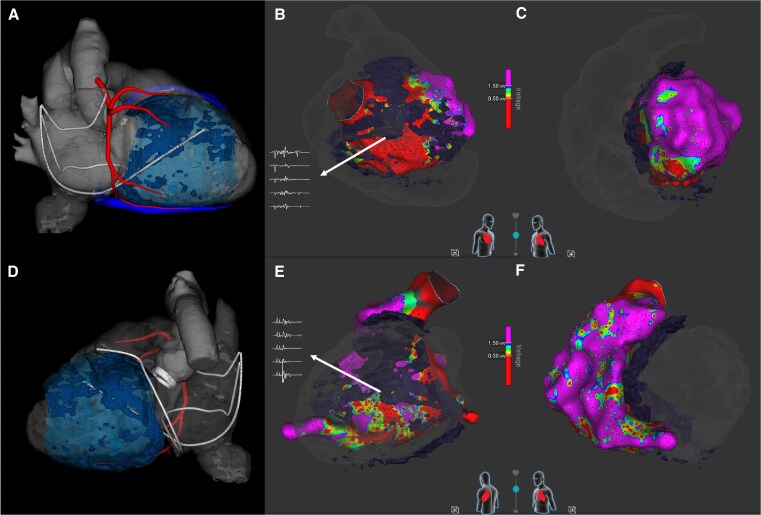
(*A* and *D*) InHEART® images derived from a cardiac computed tomography scan, where scar was defined with delayed contrast enhancement, which is depicted in light and dark blue depending on the density of the scar. (*A*) The left ventricular septum from a right anterior oblique view and (*D*) the right ventricular septum from a left posterior oblique view. The right coronary artery is shown in red, the coronary venous system in blue, and the defibrillator leads in white. The other panels show electroanatomical maps, where colour coding corresponds to voltage. Red represents low voltage (<0.50 mV) and purple represents normal voltage (>1.50 mV). An overlay of dense scar from the scan is shown in dark grey. (*B* and *E*) The left and right ventricles from the same angle as (*A* and *D*), respectively. Local bipolar electrograms in septal dense scar are shown during right ventricular apical pacing. All of the displayed electrograms are pathological and compatible with local abnormal ventricular activation. (*C* and *F*) A left anterior oblique view of both ventricles, respectively.

Endocardial ablation using a novel large footprint 9 mm lattice-tip catheter (Sphere 9, AFFERA, Medtronic) was performed (*Figure 1*). This allows for high density mapping and ablation with a single catheter, capable of delivering RFA as well as PFA. The CCT images were merged with the 3D electroanatomical map using InHEART® software, allowing for correlation of the anatomically defined substrate with the endocardial map (*Figure 2*).

The procedure was performed under general anaesthesia with a transseptal approach.

**Figure 3:**
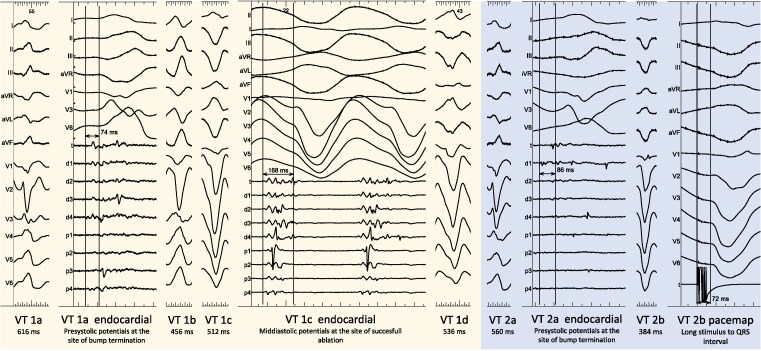
Twelve-lead ECG morphologies of the four ventricular tachycardias induced during the first procedure, as well as of the two ventricular tachycardias from the second procedure. The tachycardia cycle length is mentioned for each ventricular tachycardia. Endocardial signals are shown for ventricular tachycardias 1a, 1c, and 2a. For ventricular tachycardias 1a and 2a, presystolic potentials are shown where the bump termination of the ventricular tachycardia occurred, respectively, at the anteroseptal base of the left ventricle and the anteroseptal base of the right ventricle. For ventricular tachycardia 1c, mid-diastolic signals are shown at the apicoseptal part of the right ventricle where ablation resulted in the termination of the tachycardia (see also *Figure 4*). The best pacemap for ventricular tachycardia 2b with a long stimulus to QRS interval in the left ventricular inferior apical region is shown as well. After ablation here, the patient became non-inducible. Sweep speed for the ECGs is set at 25 mm/s. For the endocardial and pacemap tracings, it is set at 100 mm/s.

**Figure 4:**
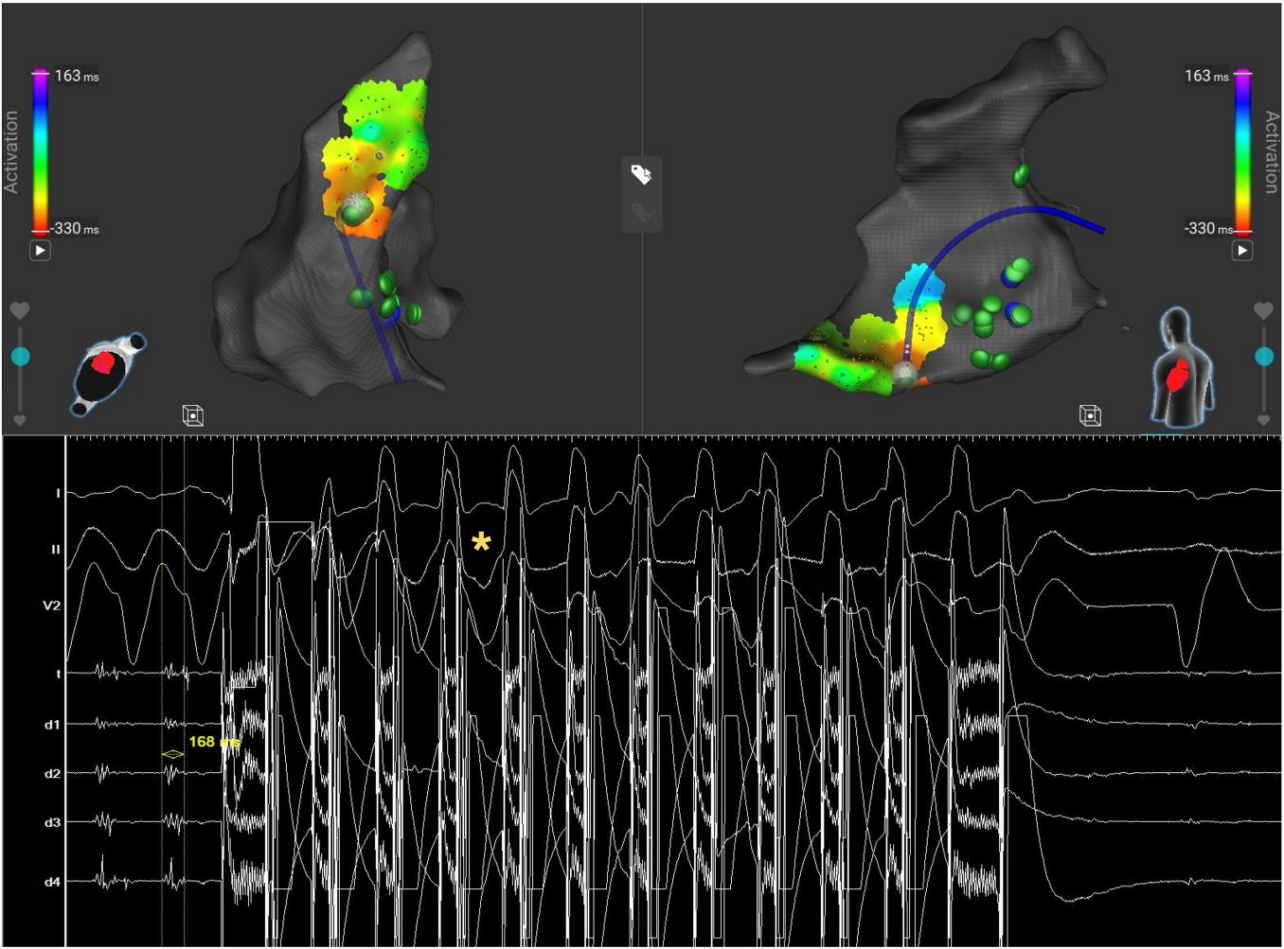
Electroanatomical map from the right ventricle showing the catheter position during ablation of ventricular tachycardia 1c from an inferior view (left) as well as from a septal view (right). The tachycardia cycle length is 512 ms. The colour coding corresponds with the local activation time of the ventricular tachycardia in relation to the reference, which is set at the peak of the QRS. Red corresponds with a local activation time of 330 ms before the reference and purple with a local activation time of 163 ms after it. The endocardial signals and three surface lead ECGs are shown on the bottom, where mid-diastolic signals preceding the QRS with 168 ms are seen (also shown in *Figure 3*). The first ablation here terminated this tachycardia. The yellow asterisk highlights the last beat of the ventricular tachycardia before termination, which is difficult to appreciate because of the artefacts during pulsed field ablation. Appropriate ablation tags are shown in green, and interrupted ablation tags are shown in blue. LAT, local activation time.

A first VT at 97 b.p.m. (VT 1a; *Figure 3*) was induced, for which activation mapping was performed in the left ventricle (LV). Pre-systolic signals were noted at the anteroseptal base of the LV, in the vicinity of the conduction system, where the VT was stopped mechanically. Substrate mapping during right ventricular (RV) apical pacing showed a zone of local abnormal ventricular activity (LAVA) at this site, which extended on both sides of the septum and towards the inferior wall, corresponding with the anatomically derived scar zone from the InHEART® scan. Three other VTs were induced during the procedure: VT 1b, which was similar to VT 1a, and VT 1c and 1d, both suggestive of an apicoseptal exit. Activation mapping of VT 1c could be partially performed, where mid-diastolic signals in the apicoseptal part of the RV were seen. Ablation here terminated this VT (*Figures 3* and *4*). Further ablation mainly targeted LAVA on both sides of the septum and the inferior wall. On the septum, two repetitive applications were performed at each site from either side of the septum. On the inferior wall, four applications were delivered at each site. In total, 99 PFA applications were done: 27 in the RV and 72 in the LV. The applications were performed according to the prespecified VT ablation protocol from Affera, where 12 trains of 125 pulses each are delivered in 5.5 s with the voltage output set at 2700 V. Catheter to tissue contact during applications was assessed by an appropriate rise in temperature. Non-inducibility was obtained at the end of the procedure.

**Figure 5:**
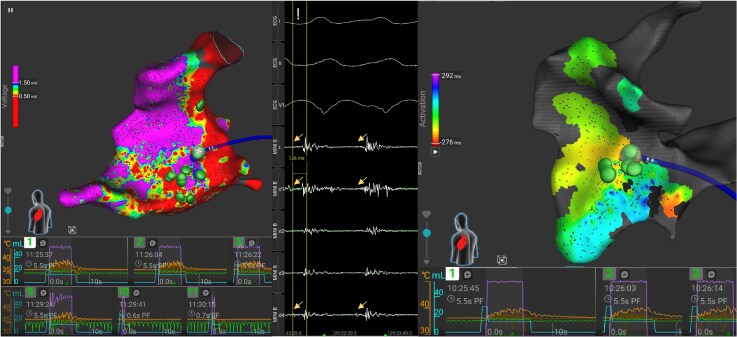
(*A*) Position of the ablation catheter during the first ablation at the anteroseptal base of the right ventricle during the first procedure. Graphical display of the ablation details for each application are shown for Lesions 1–3 and 9–11, which were all performed on the same site. For Lesions 1–3, there is an appropriate rise in temperature (orange curve), indicating a good contact with the tissue. The impedance graph (green curve) and current level graph (purple curve, expressed as a percentage of the maximum current) however show a staggered pattern, probably indicating interference with the right ventricular lead from the defibrillator (which is not shown). For Lesion 9, the curves look even more distorted. Lesions 10 and 11 were interrupted by the generator after 0.6 and 0.7 s, where a normal application takes 5.5 s. (*B*) The position of the ablation catheter during the second procedure while performing activation mapping of ventricular tachycardia 2a, where mid-diastolic potentials (highlighted by the yellow arrows) are noted in the same region that was ablated in the first procedure preceding the QRS with 126 ms (*Figure 3* shows presystolic potentials in a region close to this one). Notice the difference in ablation curves of the first three lesions done here during this procedure compared to the previous one. Also note the difference in the angle at which the catheter approaches the septum in the second procedure compared to the first procedure, probably avoiding contact with the defibrillator lead this time. Colour coding for (*A*) corresponds to voltage settings as in *Figure 2*. Colour coding in (*B*) corresponds to local activation time as in *Figure 4*. Appropriate ablation tags are shown in green, and interrupted ablation tags are shown in blue.

Two weeks later, the patient presented with a VT (VT 2a) at 107 b.p.m. lasting for more than 24 h with signs of heart failure. A redo ablation with the same system was performed. Diastolic potentials were identified in the anteroseptal base of the RV, where the VT was stopped mechanically (*Figure 3*). During the initial procedure, this area had been ablated with 2 applications per site. Retrospective analysis of those lesions revealed suboptimal ablation parameters (*Figure 5*). Ablation was repeated on both sides of the septum, this time using four applications at each site. Subsequently, another VT (VT 2b) was induced, with good pacemapping in the LV inferior apical region where a long stimulus-to-QRS interval was observed. A residual zone of LAVA persisted in this area. This region had also been targeted in the previous procedure, but again with suboptimal parameters. It was re-ablated with four applications at each site, which ultimately rendered the patient non-inducible. In total, 32 PFA applications were delivered: 12 in the RV and 20 in the LV.

At 7-month follow-up, the patient was doing well without any recurrence of VT.

## Discussion

Catheters delivering PFA have been developed for the ablation of atrial fibrillation. The design of these catheters often limited their manoeuvrability within the ventricle, e.g. due to their large size intended for single-shot pulmonary vein isolation. This hampered the application of PFA for VT. The design of the novel Sphere-9 catheter might overcome this problem, with enhanced manoeuvrability and stability in the ventricle. The first case reports and series have shown a high rate of acute non-inducibility and a low rate of recurrence during short-term follow-up.^6–9^ Whether its use in the setting of a septal substrate might improve the outcome remains unclear.

An intramural substrate is more common in the septal region, especially in non-ischaemic cardiomyopathy.^4^ This scar is more heterogenous and extends deeper into the tissue.^10^ Lesions created with conventional RFA have shown to be insufficient to reach these areas in animal models.^11^ Conversely, PFA extends deeper into the tissue, even when viable myocardium is separated from the catheter by layers of collagen and fat.^12^ This makes it an attractive tool for these patients.

In our patient, PFA served as a successful bailout strategy, even after failed SBRT, which has been considered a last resort therapy. Until now, bailout strategies consisted of bipolar ablation, intramural needle ablation, coil embolization, venous or arterial alcohol ablation, and indeed SBRT.^13^ None of these therapies are however easy to implement. It therefore highlights the potential benefit of PFA in these difficult to treat patients, especially since the use of this lattice-tip catheter is easy to integrate in a standard electrophysiology lab.

Nevertheless, our patient recurred after the first PFA procedure. During the redo procedure, we noted diastolic potentials during VT at the anteroseptal base of the RV as well as re-appearance of LAVA with an excellent pacemap in the inferior apical region (*Figure 3*). Both regions were targeted during the first procedure.

The reason for this ablation failure is probably three-fold. In the first procedure, the septum was targeted with two applications of PFA at each site, repeating two applications at the opposite side of the septum. This might result in too shallow lesions, as it has been shown in animal models that PFA is able to penetrate deeper into the tissue depending on the repetition frequency using up to four repetitions per site.^14^ On top of this, reviewing the lesions that were performed in the anteroseptal region during the first PFA procedure shows a staggered pattern of the impedance graph and of the current level graph, which might be related to interference of the catheter with the RV CRT-D lead impairing appropriate energy delivery to the tissue (*Figure 5*).

Reviewing the lesions of the first procedure in the inferior apical part shows a possible lack in contact, as can be derived from only minimal temperature rise during the applications. During the redo procedure, the applications were performed with extra focus on optimal contact, resulting in the absence of any VT during a 7-month follow-up.^15^

The recurrence of VT after the first PFA procedure could possibly have been prevented taking into account all of these considerations. Another possibility would be to combine RFA and PFA lesions, as was done in the biggest case series so far.^7^ Recently, histological analyses of this approach with another catheter showed good results.^16^

The results of this challenging case are nevertheless satisfying and promising towards a wider application of this technology in VT patients harbouring a complex intramural substrate.

## Lead author biography


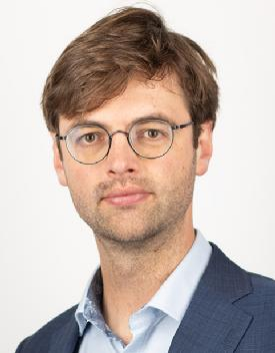


Laurens Verhaeghe is a cardiologist who completed his training at the University of Leuven (KUL, Belgium) in 2022. He then pursued a 2-year fellowship in interventional electrophysiology and pacing at Jessa Hospital in Hasselt (Belgium). Currently, he is continuing his training with a fellowship in electrophysiology at CHU Bordeaux (France), with a specific interest in ventricular arrhythmias.

**Consent:** The authors confirm that written consent for submission and publication of this case report including images and associated text has been obtained from the patient, in line with the COPE guidelines.

**Funding:** This study received financial support from the French Government as part of the ‘Investments in the Future’ program managed by the National Research Agency (ANR), grant reference ANR-10-IAHU-04.

## Data Availability

Data sharing is not applicable to this article, as no datasets were generated or analysed during the current study. The data underlying this article are available in the article.
